# Blood Transfusion Practices in Rural Hospitals: Indications, Challenges, and Practical Solutions

**DOI:** 10.7759/cureus.106752

**Published:** 2026-04-09

**Authors:** Vijay Ramesh Topno, Ghanshyam Bihari, Gaurav Mishra, Dharmendra Kumar

**Affiliations:** 1 Department of Pathology, Tata Main Hospital, Noamundi, IND; 2 Department of Ear, Nose, and Throat (ENT), Tata Main Hospital, Noamundi, IND; 3 Department of General Medicine, Tata Main Hospital, Noamundi, IND

**Keywords:** blood transfusion, cold chain, patient blood management, rural hospitals, transfusion safety

## Abstract

Blood transfusion (BT), red blood cell (RBC) transfusion, patient blood management (PBM), and transfusion-transmitted infections (TTIs) remain central to emergency and routine care in settings where access, safety, and logistics are constrained. Evidence gaps persist due to the cost and time requirements of TTI testing, limited ongoing surveillance within district systems, and diagnostic delays that shift decision-making away from laboratory thresholds. This narrative review aims to synthesise BT indications, operational challenges, and practical solutions relevant to decentralised and remote hospital environments. A structured literature synthesis was conducted using databases including PubMed, Scopus, and Google Scholar, applying predefined search terms related to BT practices, rural healthcare, and transfusion safety for studies published between 2015 and 2025; studies were selected based on relevance to clinical indications, logistical challenges, and safety outcomes in resource-limited settings. Key outcomes included comparative transfusion threshold findings in haemoglobinopathies and sepsis, the feasibility of universal-donor products in trauma, and the role of cold-chain and storage systems in maintaining supply integrity. Practice implications include prioritising physiological criteria for urgent BT when laboratory access is delayed, while strengthening governance through clinical audits and point-of-care electronic verification. The findings support integrated transfusion pathways that combine decentralised preparedness with mandatory pathogen screening and standardised monitoring frameworks. The central takeaway is that safe rural BT systems require coordinated investment in supply resilience, workforce competency, and technology-enabled verification, rather than reliance on isolated interventions.

## Introduction and background

Chronic shortages of safe blood in developing countries contribute to preventable morbidity and mortality from otherwise treatable conditions and injuries [1]. Blood is a finite and expensive resource, constrained by low donation rates and poor stock management, which limit availability in healthcare settings worldwide [2]. In parts of West Africa, higher rates of seroprevalence of transfusion-transmissible infections are associated with increased risk in blood transfusion (BT) and greater demands on screening systems [3]. In austere and remote settings, long supply chains and delayed resupply limit timely access to blood for patients with severe haemorrhage [4]. Military and emergency trauma care emphasises the importance of rapid BT support in conjunction with damage control surgery to improve survival in life-threatening bleeding [5]. Despite these recognised challenges, there remains limited synthesis of evidence integrating clinical decision-making, logistical constraints, and safety governance within rural and resource-limited transfusion systems. This narrative review aims to synthesise current evidence on BT practices, operational challenges, and safety considerations in rural and resource-limited hospital settings, with a focus on improving clinical decision-making and system resilience.

Preparedness for BT is a critical but challenging issue for isolated facilities with limited infrastructure and workforce capacity [6]. In the public health sector of developing countries such as South Africa and India, RBC transfusion remains a key therapeutic intervention for large patient populations receiving state care [7]. Nurses are integral to all stages of the transfusion process, from request initiation to monitoring patients for adverse reactions [8,9]. Continuous vigilance for infections such as HIV and syphilis is necessary to ensure recipient safety [10]. These workforce and infrastructure constraints further compound the challenges of delivering safe and timely transfusion care in decentralised settings.

Maintaining contingency reserves of compatible blood and ensuring appropriate storage conditions are essential to sustaining an uninterrupted blood supply during disruptions [4]. Clinical audits help determine whether BT practices comply with established guidelines and identify knowledge gaps among staff [7]. Rural hospitals experience logistical challenges in screening for transfusion-transmissible infections, often resulting in delays in accessing safe blood [1]. Limited data on the burden of transfusion-transmissible infections among donors in district-level surveillance systems restrict accurate assessment of local risk; although nucleic acid testing offers high sensitivity, its implementation in rural settings is constrained by cost and infrastructure, making serological screening and strengthened donor selection more practical risk-reduction strategies [9]. These limitations highlight gaps between recommended transfusion standards and their practical implementation in rural healthcare environments.

Decentralised blood systems can enhance local self-sufficiency, as extended transport routes often delay care [6]. Strict donor selection and adherence to screening protocols minimise the risk of infection for recipients [10]. Resource-limited settings must prioritise high-impact screening and prevention strategies to optimise the use of scarce resources [3]. Ongoing research continues to evaluate the effectiveness of various blood products in prolonged care settings to optimise BT practices [5]. Existing evidence also examines nurses' bedside knowledge and practical performance related to safe BT within structured hospital safety frameworks [8]. This review therefore examines how clinical thresholds, supply logistics, and safety governance can be integrated to improve transfusion practice in rural healthcare environments.

Objectives of the review

This narrative review was designed to evaluate the quality of resuscitation using various blood products in models that simulate prolonged care. It also aims to assess the level of knowledge regarding safe transfusion practices through bedside and practical clinical performance of nurses.

Methodology

This narrative review was conducted using a structured literature search of electronic databases, including PubMed, Scopus, and Google Scholar. Search terms included combinations of ‘blood transfusion,’ ‘rural hospitals,’ ‘transfusion safety,’ ‘patient blood management,’ and ‘transfusion-transmitted infections.’ The search strategy combined Medical Subject Headings (MeSH) and free-text terms using Boolean operators (AND, OR) to capture relevant studies on blood transfusion practices, rural healthcare delivery, transfusion safety, and patient blood management. Studies published between 2015 and 2025 were considered. Inclusion criteria comprised peer-reviewed articles addressing transfusion indications, safety practices, logistics, and outcomes in resource-limited or rural settings. Exclusion criteria included non-English publications, case reports with limited generalizability, and studies lacking relevance to transfusion practices. Study selection was performed through a stepwise screening process, including title and abstract review followed by full-text assessment to determine eligibility based on predefined criteria. Articles were screened based on title and abstract, followed by full-text review to assess eligibility. Given the narrative nature of this review, studies were critically appraised for relevance, methodological rigor, and applicability to rural or resource-limited healthcare settings, rather than through formal risk-of-bias assessment tools. Relevant data were synthesised thematically across clinical, logistical, and safety domains to provide an integrated interpretation of the available evidence.

## Review

Clinical indications and transfusion thresholds in high-acuity and resource-limited settings

In patients with haemoglobinopathies and acute anaemia, restrictive transfusion strategies and a liberal transfusion threshold (10 g/dL) do not significantly affect mortality or recurrent infarction within 30 days [11]. Among extremely low birth weight infants, there is no significant survival or neurodevelopmental benefit associated with higher haemoglobin thresholds at two years [12]. Emulated target trial data suggest that lowering transfusion thresholds from 10 g/dL to 7 g/dL is associated with an increased risk of early mortality and recurrent infarction [13]. In adult sepsis, lower 28-day mortality has been associated with transfusion at haemoglobin levels between 7 and 9 g/dL compared with levels below 7 g/dL [14]. Collectively, these findings indicate that transfusion thresholds must be individualised based on clinical context rather than applied uniformly across patient populations.

**Figure 1:**
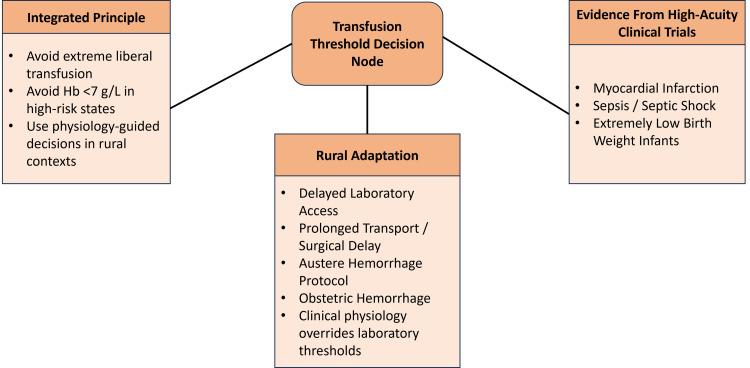
Context-Responsive Transfusion Decision Framework in High-Acuity and Rural Settings Created by authors using Microsoft PowerPoint

In rural referral centres, transfusion decisions are often based on clinical signs such as pallor, active bleeding, and signs of shock due to delays in laboratory reporting [1]. This reliance on clinical assessment reflects the limited availability of timely laboratory diagnostics in resource-constrained settings. Balanced component resuscitation strategies have been shown to improve haemodynamic stability in life-threatening haemorrhage [4,5]. Obstetric haemorrhage guidelines recommend transfusion at higher haemoglobin thresholds when there is evidence of impaired tissue perfusion [7]. These approaches highlight the need to integrate physiological indicators with evidence-based thresholds in rural transfusion practice. A context-adapted transfusion decision framework that combines trial-based haemoglobin thresholds with physiologically guided triggers, tailored to rural healthcare constraints, is illustrated in Figure 1.

Blood product utilisation, demand drivers, and transfusion safety strategies

Acute or delayed haemorrhage following surgical or endoscopic procedures may require transfusion if haemoglobin levels fall below approximately 7.0 g/dL in patients without haemodynamic instability or significant comorbidities [15]. Timely assessment of blood loss in rural hospitals is essential, particularly where advanced interventional support is limited [16,17]. Trauma data indicate that approximately 80% of severely injured patients require transfusion of packed RBCs, 69% require plasma, and 12% require cryoprecipitate during resuscitation [18]. These findings highlight the substantial and variable demand for blood components across high-acuity clinical scenarios.

Blood supply deficits remain prevalent in low-income settings, with some regions meeting less than 42% of clinical demand [1]. Remote services may utilise community-based donor systems to enhance local availability, along with strategic long-term storage and mandatory pathogen screening to strengthen the safety and continuity of inventory [4,10]. Addressing shortages requires decentralised collection systems, adequate preservation infrastructure, and robust transfusion governance frameworks [1]. These strategies are essential to balance clinical demand with constrained supply in rural healthcare systems.

Group A emergency release plasma is non-inferior to group AB plasma in terms of thromboembolic events, with no increase in haemolytic reactions observed in trauma patients requiring massive transfusion [19]. Prehospital and in-hospital resuscitation using low-titre group O whole blood is safe, feasible, and associated with a reduced requirement for RBC components within the first 24 hours compared with standard care [20]. In selected severe cases of sickle cell disease, RBC exchange therapy reduces haemoglobin S levels to below 30% prior to stem cell mobilisation [21]. These emergency compatibility strategies support rapid and flexible transfusion responses in critical care settings.

Bedside electronic transfusion checks using barcode systems help reduce labelling and patient identification errors [22]. Nucleic acid testing, although highly sensitive for detecting infections, is limited in resource-constrained settings due to cost and infrastructure requirements [23]. Sensitive serological screening for transfusion-transmitted infections remains critically important [10]. Knowledge gaps among nursing staff regarding cross-matching times and appropriate storage temperatures have been reported [8]. Post-transfusion haemoglobin reassessment within 24 hours supports adherence to clinical protocols and appropriate transfusion practices [13]. Structured emergency protocols for un-crossmatched group O blood improve safety in the management of acute haemorrhage [4]. An integrated approach combining screening, monitoring, workforce training, and electronic verification is essential to strengthen transfusion safety systems.

Massive haemorrhage management and risk-balanced transfusion protocols

In major trauma haemorrhage, the addition of viscoelastic haemostatic assays to established protocols does not reduce massive transfusion rates at 24 hours compared with conventional coagulation testing [24]. Management of severe haemorrhage across trauma and surgical settings relies on standardised transfusion triggers that balance timely resuscitation with the risk of post-transfusion complications [2,7,18,24]. Abdominal radical hysterectomy for invasive cervical cancer carries a significant risk of perioperative haemorrhage, with approximately 15% of patients requiring transfusion between 2010 and 2019 [2]. RBC transfusion in obstetric haemorrhage is indicated when haemoglobin levels range between 6-10 g/dL or when there are clinical signs of inadequate perfusion [7]. These findings highlight the need for context-specific transfusion thresholds across different high-risk clinical scenarios.

Cohort studies demonstrate a dose-dependent association between the number of transfused blood units and an increased risk of nosocomial infection during hospitalisation [18]. Group A emergency release plasma is a safe and effective alternative to group AB plasma for massive transfusion, particularly when universal donor supplies are limited [19]. Contemporary massive transfusion protocols increasingly incorporate the use of universal donor products and advanced coagulation assessment tools while minimising delays associated with laboratory processing, thereby improving overall management of end-organ dysfunction and infection risk [25-27]. These protocol-driven approaches aim to optimise outcomes while mitigating transfusion-related complications. Evidence indicates that viscoelastic haemostatic assay (VHA)-augmented protocols do not provide a significant 24-hour benefit over conventional testing in trauma settings, as summarised in Table 1.

**Table 1:** Inventory Optimisation and Wastage Reduction in Low-Volume Hospitals VHA: Viscoelastic Haemostatic Assay; MHPs: Massive Haemorrhage Protocols; MODS: Multiple Organ Dysfunction Syndrome; Hb: Haemoglobin; TBSA: Total Body Surface Area

Trial Name	Therapeutic Intervention	Condition	Cohort Size	Primary Endpoint	Secondary Endpoints	Key Efficacy Results	Safety Findings	Reference
ITACTIC	VHA-augmented MHPs vs. Conventional Coagulation Tests	Major trauma haemorrhage	396	Alive and free of massive transfusion at 24 h	Mortality, MODS, thromboembolism, ventilator-free days	No difference at 24 h; 28-day mortality similar	Infection 31%, thromboembolism 14%, acute lung injury 11%	[21]
TRIBE	Restrictive (Hb 7–8 g/dL) vs. Liberal (Hb 10–11 g/dL)	Adult burns ≥20% TBSA	303	Severity of multiple organ dysfunction	Wound healing, ventilation duration, and mortality	Storage age had no impact on MOD, healing, or mortality	Ventilation was weakly predicted by storage age	[27]

Cold chain integrity, storage systems, and blood product preservation

In massively transfused burn patients, a mean RBC storage age of approximately 26 days does not significantly affect organ dysfunction severity or in-hospital mortality [26]. Active perioperative warming measures, such as fluid warmers and thermal blankets, maintain core temperature above 36°C and reduce blood loss and transfusion requirements in major surgery [27,28]. During intraoperative blood salvage, suction pressures exceeding 200 mmHg increase haemolysis and erythrocyte damage [29]. These findings highlight the importance of temperature control and mechanical factors in preserving blood product integrity during transfusion and surgical care.

The immediate availability of un-crossmatched group O blood in emergency settings is often limited due to challenges in maintaining adequate inventory and high clinical demand [30]. Strategic −80°C storage systems enable long-term preservation and transport of blood products in remote environments [4]. Clearly defined cold chain protocols between central blood banks and peripheral units are essential in decentralised systems [6]. Transport delays in mandatory screening processes further restrict timely access to safe blood in remote settings [1]. These logistical constraints underscore the critical role of coordinated storage and transport systems in ensuring timely and safe transfusion.

**Figure 2:**
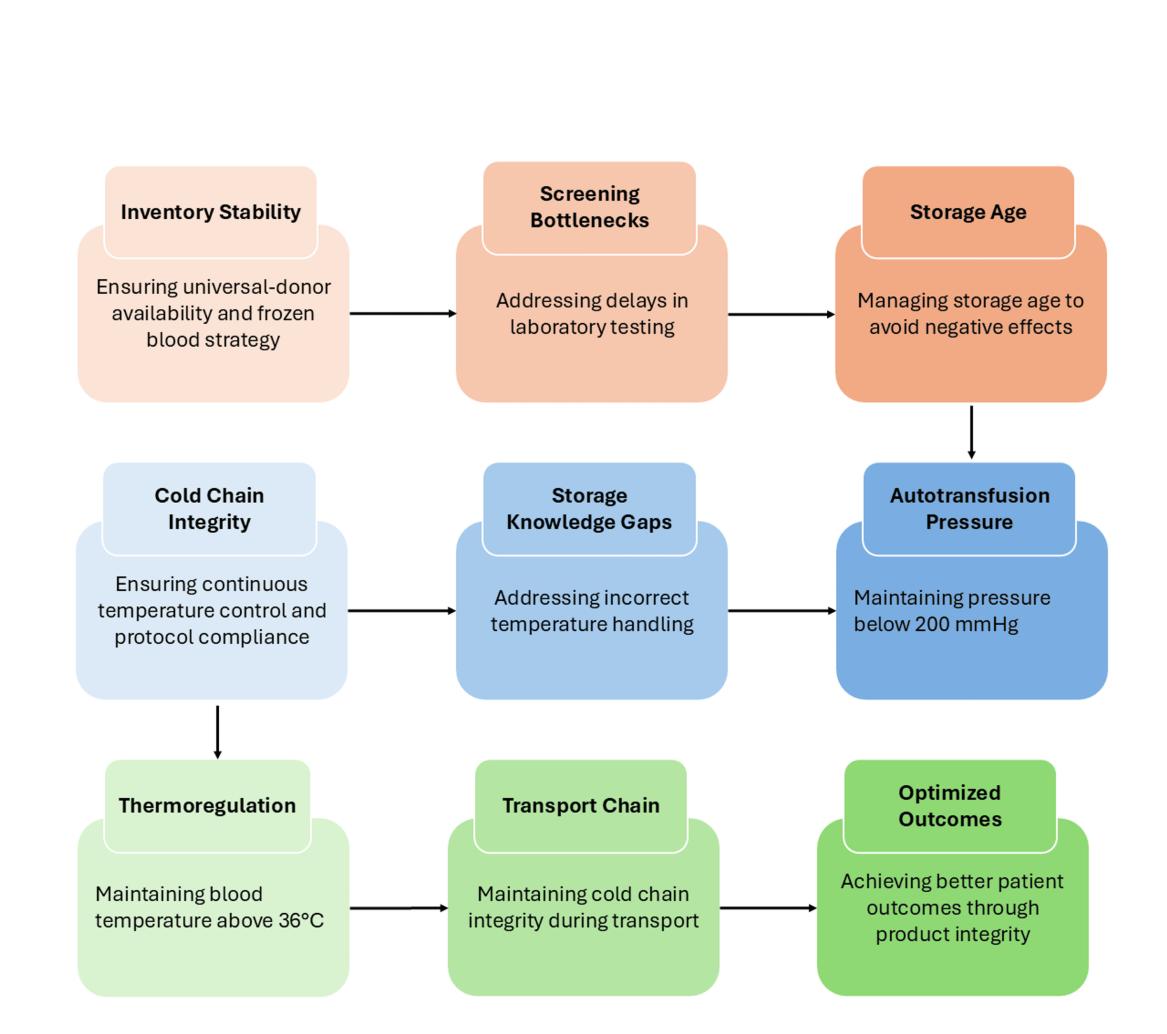
Cold Chain Integrity and Product Preservation Pathway in Rural and High-Acuity Settings Created by authors using Microsoft PowerPoint

Staffing limitations, particularly knowledge gaps related to storage temperatures and return procedures, contribute to blood product wastage [8]. Robust supply chain governance ensures an optimal balance between availability, preservation technologies, and patient thermoregulation [4,5]. Strengthening workforce competency alongside infrastructure is therefore essential to maintain cold chain integrity and minimise resource loss. Figure 2 illustrates the pathway of cold chain preservation from inventory control to optimised patient outcomes.

Table 2 shows the safety and feasibility of emergency group A plasma and low-titre O whole blood strategies in massive transfusion care.

**Table 2:** Pre-Transfusion Testing Pathways and Emergency Compatibility Practices PROPPR: Pragmatic, Randomized Optimal Platelet and Plasma Ratios; ERP: Emergency Release Plasma; SIRS: Systemic Inflammatory Response Syndrome; AKI: Acute Kidney Injury; HR: Hazard Ratio; POWeR-MTP: Pragmatic O-Positive Whole Blood Randomization in Massive Transfusion Protocol; PRBC: Packed Red Blood Cells; TRALI: Transfusion-Related Acute Lung Injury

Trial Name	Therapeutic Intervention	Condition/Disease	Cohort Size	Primary Endpoint	Secondary Endpoints	Key Efficacy Results	Safety Findings	Reference
PROPPR	Group A ERP vs. Group AB ERP; 1:1:1 vs. 1:1:2 ratios	Severe trauma requiring massive transfusion	680 (584 analysed)	30-day mortality	Complications, SIRS, AKI, infection, thromboembolism	Mortality similar; thromboembolic HR 0.52	No hemolytic reactions; febrile reactions in the AB group	[19]
POWeR-MTP	Low-titre O-positive whole blood vs. component therapy	Traumatic haemorrhagic shock	199	Transfusion requirement (≥24 h survivors)	All-cause mortality	Mortality similar; fewer PRBC units trend	Rare TRALI; anti-D alloimmunization 7.8%	[28]

Safety, workforce competency, and governance in transfusion practice

Accurate assessment of patients requiring BT relies on systematic clinical evaluation and standardised documentation to ensure consistent decision-making among healthcare providers [31]. Adherence to established transfusion guidelines and institutional protocols is essential to maintain patient safety, ethical practice, and accountability within rural hospital settings [32]. Laboratory screening of donor blood must be performed using validated procedures to detect transfusion-transmissible infections and ensure compatibility prior to administration [33]. Proper record-keeping, traceability of blood units, and post-transfusion monitoring are critical to enhancing haemovigilance and quality assurance in transfusion services [34]. These components form the foundation of safe and accountable transfusion practice in resource-limited settings.

Transfusion training programmes for nursing staff in rural oncology and general medical hospitals should include key competencies such as appropriate storage temperatures, compatibility testing methods, permissible cross-matching times, and mandatory pathogen screening to ensure safe administration of blood products [8]. Clinical audits in district hospitals have shown that junior medical staff, particularly interns, often demonstrate lower levels of knowledge in the appropriate selection and ordering of blood components compared with senior clinicians, highlighting the need for structured educational interventions [7]. Workforce limitations, particularly inadequate laboratory staffing, limit the ability to use haemoglobin levels as a timely indicator for initiating emergency resuscitation [1]. The implementation of bedside electronic verification systems requires a phased approach, including simulation-based training and mandatory competency assessments for clinical personnel [22]. Strengthening workforce competency through training, audit, and digital verification is essential to maintaining operational reliability in high-acuity environments.

Therapeutic plasma exchange for severe vasculitis requires close monitoring for bleeding and infectious complications that may necessitate urgent support with BT [35]. In paediatric cardiac care, major bleeding is defined as overt haemorrhage, a significant decrease in haemoglobin level, or the need for rescue transfusion [36]. Intensive oncology treatments require monitoring for myelotoxicity and infections, as these factors influence survival and transfusion requirements [37]. Long-term follow-up is necessary to identify delayed immunologic complications, such as autoimmune haemolytic anaemia, following advanced therapies [38]. These examples highlight the importance of continuous monitoring for treatment-related risks across diverse clinical contexts.

ABO-incompatible transfusion events commonly result from errors in patient identification and sample labelling at the point of care [22]. Group A emergency release plasma is a safe alternative to group AB plasma in massive transfusion protocols [39]. Knowledge gaps in blood storage practices contribute to both wastage and increased safety risks [8]. Increased exposure to transfused blood products is associated with a higher risk of nosocomial infection in trauma patients [18]. Integrated electronic verification systems and structured monitoring protocols are essential to reduce incompatibility errors, haemorrhagic complications, and delayed organ dysfunction.

Nursing staff are central to bedside monitoring for the early recognition and management of transfusion reactions [8]. The use of frozen blood products in high-acuity settings has demonstrated low rates of adverse reactions when appropriate storage standards are maintained [40]. Rural whole blood programmes rely on clinically defined triggers for emergency resuscitation in resource-constrained environments [6]. Prehospital administration of low-titre group O whole blood has been shown to be safe and associated with low complication rates in trauma care [18]. Structured monitoring frameworks are critical to differentiating mild allergic reactions from severe haemorrhagic or organ-threatening complications, thereby improving patient safety.

Patient blood management and allogeneic exposure reduction strategies

**Figure 3:**
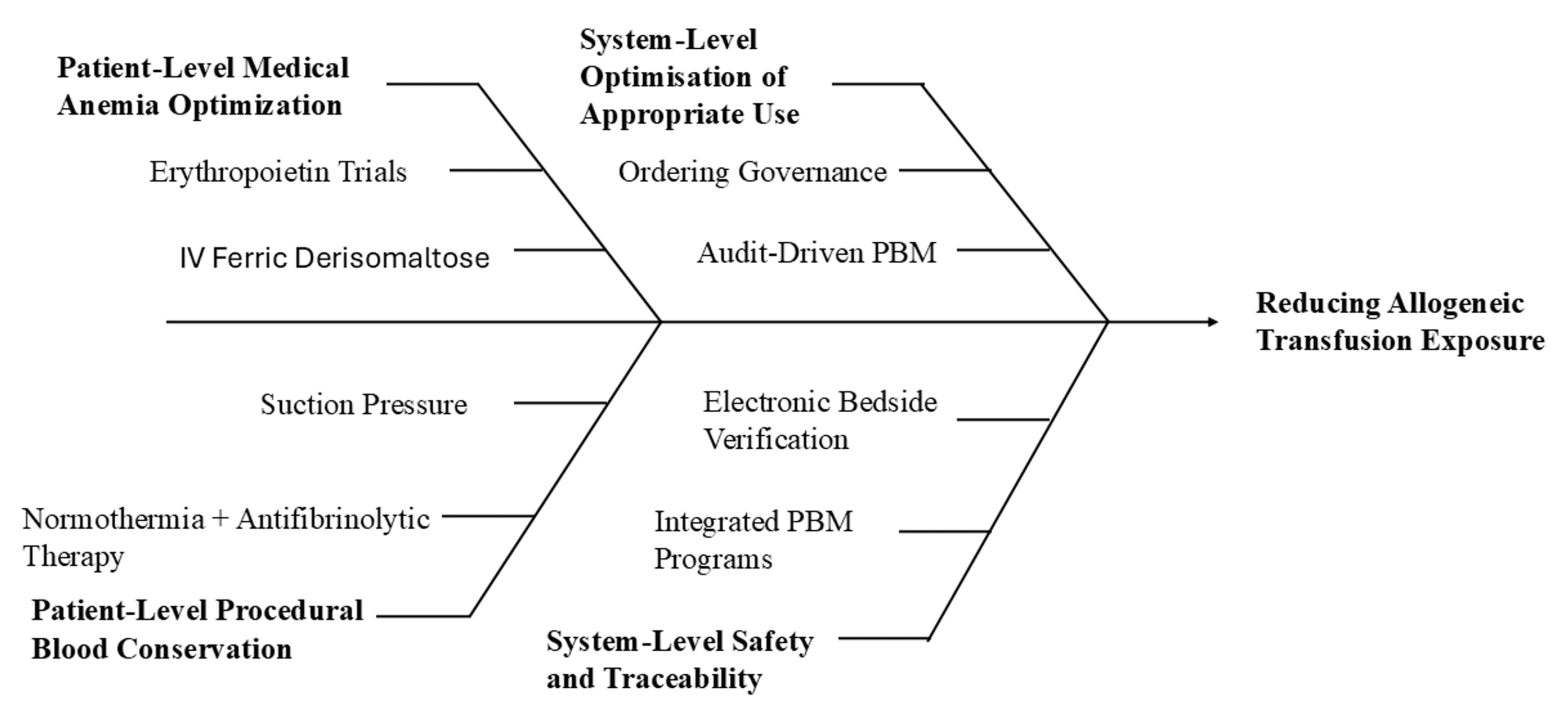
Patient Blood Management (PBM) and Allogeneic Exposure Reduction Strategies Created by authors using Microsoft PowerPoint

Intravenous iron therapy has been associated with improved clinical outcomes and reduced hospitalisation in selected patient populations, supporting its role in anaemia optimisation strategies within patient blood management (PBM) [37]. In perioperative care, maintaining suction pressure below 200 mmHg during autotransfusion reduces haemolysis and erythrocyte damage, contributing to safer blood conservation practices [25]. Electronic bedside verification systems enhance patient identification and blood unit traceability, reducing transfusion errors [20]. Blood resource optimisation within district hospitals remains constrained by limited clinical audit implementation and gaps in transfusion knowledge among junior clinicians [7]. Effective PBM integrates anaemia correction, procedural blood conservation, governance structures, and electronic verification systems to reduce unnecessary allogeneic transfusion exposure. A quadrant-based PBM framework integrating these components is illustrated in Figure 3.

Table 3 shows the intravenous iron benefit in heart failure and the impact of pressure-controlled autotransfusion on haemolysis risk in surgical patients.

**Table 3:** Patient Blood Management Strategies and Autotransfusion Outcomes FDI: Ferric Derisomaltose; Hb: Haemoglobin; MLwHF: Minnesota Living with Heart Failure Questionnaire; 6MWD: Six-Minute Walk Distance; CV: Cardiovascular; RR: Relative Risk; CI: Confidence Interval; MCV: Mean Corpuscular Volume; Hct: Haematocrit

Trial Name	Therapeutic Intervention	Condition/Disease	Cohort Size	Primary Endpoint	Secondary Endpoint(s)	Key Clinical Efficacy Results	Safety and Adverse Events	References
IRONMAN (NCT02642562)	Intravenous ferric derisomaltose (FDI) vs. usual care	Heart failure with iron deficiency	1137	Recurrent heart failure hospitalisation or cardiovascular death	Hb, MLwHF, 6MWD, CV and all-cause mortality	Composite reduction RR 0.82 (95% CI 0.66–1.02, P=0.070); improved MLwHF physical domain (P=0.0071)	Not Reported	[37]
Autotransfusion pressure control system study	Autotransfusion with pressure-control (negative pressures: 100–300 mmHg)	Orthopaedic and neurosurgical patients with blood loss >400 mL	50	Hb, Hct, MCV, standardised plasma-free Hb, haemolysis rate	Erythrocyte morphology, surgeon satisfaction	Haemolysis increased exponentially with pressure; abnormal erythrocytes increased	Risk of macroscopic haemoglobinuria/renal failure at high pressures	[25]

Technology-enabled governance and long-term monitoring in transfusion practice

Long-term therapeutic surveillance and outcome monitoring remain important in transfusion practice; however, their implementation in rural settings is often limited by infrastructure and resource constraints. These limitations restrict the ability to establish consistent monitoring benchmarks and evaluate long-term transfusion outcomes. In district hospitals, clinical audits reinforce adherence to transfusion guidelines and help identify knowledge gaps among clinicians [7]. Electronic verification of bedside transfusion improves traceability and reduces errors in patient identification [20]. Technology-driven decision support systems, including structured electronic workflows and viscoelastic testing where available, can reduce variability in care and minimise preventable errors [20]. Together, these tools support standardisation of practice and strengthen governance frameworks in resource-limited settings. Blood safety governance remains challenging in such environments due to delays in pathogen screening associated with reliance on centralised laboratory systems [1]. Addressing these constraints requires integration of digital systems, audit mechanisms, and decentralised diagnostic support to improve the reliability and safety of transfusion services.

Limitations and future directions

Work in this field is hampered by several limitations, including the high cost and time requirements associated with transfusion-transmissible infection testing in rural hospitals. Evidence synthesis is further limited by the absence of sustained surveillance systems and reliable data on infection burden among donor populations in district hospitals. Additionally, the applicability of existing evidence is constrained, as most transfusion studies are conducted in well-resourced settings with reliable laboratory infrastructure, advanced diagnostics, and established haemovigilance systems, which are often unavailable in rural healthcare environments. The implementation of laboratory-driven transfusion strategies is restricted by workforce limitations, particularly inadequate laboratory staffing, which limits the use of haemoglobin measurements for timely decision-making in emergency resuscitation. Rural hospitals frequently face delayed diagnostics, constrained monitoring capacity, infrastructure limitations, and reliance on less sensitive screening methods, all of which may compromise the safe implementation of standard transfusion protocols. Safety improvement efforts remain vulnerable to human error at the point of care, particularly in patient identification and sample labelling, contributing to ABO-incompatible transfusion events. These contextual differences may reduce the generalisability of current transfusion strategies and highlight the need for context-specific adaptations tailored to rural practice.

Future work should focus on decentralised systems that enhance local self-sufficiency in blood availability, particularly in settings where long transport distances delay access to centralised blood supplies. Research should prioritise the identification of targeted screening and prevention strategies to enable efficient resource allocation in areas with high prevalence of blood-borne infections. Quality improvement initiatives should emphasise the expanded use of clinical audits to identify knowledge gaps and improve adherence to transfusion guidelines in district hospitals. Technology-based decision support systems should be evaluated based on their ability to standardise clinical workflows and reduce preventable errors in high-acuity care settings.

## Conclusions

This review synthesises current evidence on transfusion practices in rural hospitals to clarify how clinical thresholds, supply logistics, and safety governance intersect in resource-constrained environments. Across high-acuity conditions, including haemoglobinopathies, sepsis, trauma, and obstetric haemorrhage, transfusion strategies require careful calibration to balance restrictive triggers with the risks of delayed intervention. In rural settings, diagnostic limitations and prolonged transport times necessitate reliance on physiological indicators of shock alongside standardised massive transfusion protocols. Demand for specialised blood components is driven by trauma resuscitation, plasma exchange, and surgical bleeding, while supply resilience depends on decentralised collection models, storage capacity, and rigorous pathogen screening. Cold chain preservation, temperature control, and pressure-regulated autotransfusion are critical to maintaining product integrity. Workforce competency, bedside electronic verification, and clinical audit systems are essential safeguards against preventable identification errors and incompatible transfusions. PBM strategies, including targeted iron therapy and controlled autotransfusion, support the reduction of unnecessary allogeneic exposure. Long-term therapeutic surveillance and technology-enabled decision support further strengthen governance frameworks. Sustainable rural transfusion systems require integrated investment in logistics, training, monitoring, and evidence-based clinical protocols to ensure safe and equitable care delivery.
